# FBXO45 levels regulated ferroptosis renal tubular epithelial cells in a model of diabetic nephropathy by PLK1

**DOI:** 10.1515/med-2024-0971

**Published:** 2024-05-31

**Authors:** Bingming Zhu, Yongxuan Hu, Ruishan Wu, Quan Yu, Wangrong Wen

**Affiliations:** Department of Clinical Laboratory, The First Affiliated Hospital of Jinan University, Guangzhou, 510630, China; Department of Dermatology and Venereology, The 3rd Affiliated Hospital of SouthernMedical University, Guangdong Provincial Key Laboratory of Bone and Joint Degeneration Diseases, Guangzhou, 510600, China; NHC Key Laboratory of Male Reproduction and Genetics, Guangdong Provincial Reproductive Science Institute (Guangdong Provincial Fertility Hospital), Guangzhou, 510600, China; Medical Experimental Research Center, School of Medicine, Jinan University, Guangzhou, Guangdong, 510630, China; Clinical Laboratory Center, The Affiliated Shunde Hospital Of Jinan University, Foshan, Guangdong, 528305, China

**Keywords:** FBXO45, diabetes nephropathy, PLK1

## Abstract

**Objective:**

This research aims to investigate the role and underlying biological mechanism of FBXO45 in regulating ferroptosis of renal fibrocytes in a diabetic nephropathy (DN) model.

**Methods:**

C57BL/6 mice were fed with a high-fat diet and injected with streptozotocin to induce diabetes. Human renal glomerular endothelial cells stimulated with d-glucose.

**Results:**

Serum FBXO45 mRNA expression was found to be down-regulated in patients with DN. There was a negative correlation between the expression of serum FBXO45 mRNA and serum α-SMA, Collagen I, and E-cadherin mRNA in patients with DN. Additionally, the expression of serum FBXO45 mRNA showed a negative correlation with blood sugar levels. Based on a 3D model prediction, it was observed that FBXO45 interacts with polo-like kinase 1 (PLK1) at GLY-271, ILE-226, GLY-166, LEU-165, ARG-245, and ASN-220, while PLK1 interacts with FBXO45 at TYR-417, ARG-516, HIS-489, TYR-485, GLN-536, and ARG-557. This interaction was confirmed through immunoprecipitation assay, which showed the interlinking of FBXO45 protein with PLK1 protein.

**Conclusions:**

These findings indicate that FBXO45 plays a role in mitigating ferroptosis in DN through the regulation of the PLK1/GPX4/SOX2 pathway. This highlights the potential of targeting FBXO45 as a therapeutic approach to ameliorate ferroptosis in DN.

## 1 Introduction

Diabetic nephropathy (DN) is the most prevalent and severe complication of diabetes, accounting for a significant proportion of chronic microvascular complications. The incidence and mortality rates of DN are increasing among diabetes patients globally [1]. In the United States, there has been a substantial rise in the number of diabetes patients developing end-stage renal disease, while in China, the prevalence and incidence of DN have also witnessed a sharp increase over the past decade [2]. According to the 2019 report by the International Diabetes Federation, it is projected that the number of diabetes patients will reach 578 million by 2030, with approximately 20–40% of them developing DN, ultimately leading to the development of chronic renal failure [3]. Consequently, there is an urgent need to investigate the underlying mechanisms and treatment strategies for DN [4].

The onset of DN can be insidious, often presenting with negative urinary albumin levels in the early stages, making it difficult to detect kidney damage symptoms [5]. However, if left untreated, proteinuria will progressively develop [5]. Once proteinuria reaches a significant level, the risk of progressing to end-stage renal failure becomes approximately 14 times higher compared to other kidney diseases, posing a substantial threat to patients and potentially leading to fatal outcomes in severe cases [6]. Consequently, early diagnosis, prevention, and intervention strategies to delay the onset and progression of DN are crucial in enhancing both the quality of life and survival rates among individuals with diabetes [7].

Renal fibrosis (RF) represents a shared mechanism through which various chronic kidney diseases progress to end-stage renal failure. Therefore, the exploration of treatment strategies targeting RF has become a central focus in nephrology research [8,9]. RF primarily involves an abnormal accumulation of extracellular matrix (ECM), particularly collagen, within the kidney, leading to significant impairment of renal structural domains [10]. Consequently, inhibiting ECM synthesis within the kidney emerges as a critical approach for the treatment of RF.

Recent studies have elucidated the involvement of ferroptosis in the pathogenesis of DN. Ferroptosis, a recently identified form of regulated cell death, is characterized by its reliance on iron and reactive oxygen species (ROS) [11]. The mounting body of evidence supports the notion that ferroptosis plays a prominent role in the development of DN, further underscoring its significance [12]. Furthermore, in recent years, research has demonstrated the potential of certain active components found in traditional Chinese medicine to ameliorate DN progression through targeted modulation of ferroptosis [13].

Previous investigations have revealed that elevated levels of integrin-linked kinase can promote the proliferation and clonogenicity of bladder cancer cells. Moreover, the anticancer drug docetaxel has been shown to reduce apoptosis and increase invasion of bladder cancer cells, concomitant with an upregulation of ROS expression [14,15]. Polo-like kinase 1 (PLK1), a pivotal regulator of cell mitosis, participates in various biological processes, including cell cycle control and cytokine secretion [16]. It plays a critical role in initiating, maintaining, and completing mitosis. Studies have demonstrated that PLK1 is highly expressed in several malignant tumors such as melanoma, cutaneous Merkel cell carcinoma, pancreatic cancer, and liver cancer, with its expression level being closely associated with prognosis in some cancers [17,18]. Suppression of PLK1 expression through diverse approaches significantly diminishes cell proliferation while promoting tumor apoptosis [14].

FBXO45, a constituent protein of the SCF complex, belongs to the F-box protein family [19]. Its primary role is to facilitate the ubiquitination and subsequent degradation of substrate proteins. Despite being one of the members, there is currently limited research available regarding its specific function [14]. Situated on the human chromosome as an aging gene, FBXO45 acts as an inhibitor of cell proliferation and hinders progression through the G1 phase of the cell cycle [20]. Conversely, studies have indicated that FBXO31, another member of the F-box protein family, exhibits reduced expression in esophageal cancer and is implicated in tumor initiation and progression [21]. The objective of this study is to elucidate the role and underlying biological mechanism of FBXO45 in modulating ferroptosis in renal fibrocytes using a DN model.

## 2 Materials and methods

### 2.1 Patients

Patients with DN and normal healthy volunteers were obtained at The First Affiliated Hospital of Jinan University. Serum samples were collected and immediately stored at −80°C. The written informed consents were obtained from all the subjects and this study was approved by the Ethics Committee of The First Affiliated Hospital of Jinan University. The clinical parameters of the DN patients and healthy controls are shown in Table 1.

**Table 1:** The clinical parameters of the DN patients and healthy controls

Group	Normal	DN patients
Number	12	12
Weight (kg)	61.67 ± 11.20	74.50 ± 11.62
BMI	22.05 ± 1.25	25.20 ± 1.63
Age (year)	46.83 ± 4.91	49.25 ± 4.33
Sex (male/female)	6/6	6/6
MAP (mm Hg)	103.83 ± 6.25	102.10 ± 4.90
LDL-C (mmol/L)	2.29 ± 0.17	2.30 ± 0.17
BUN (mmol/L)	5.12 ± 0.68	8.76 ± 0.98

### 2.2 Animal care model

C57BL/6 mice (male, 4–5 weeks, 17–19 g) were fed with a high-fat diet for 12 weeks and then injected with STZ (30 mg/kg of streptozotocin; Sigma, St Louis, MO, USA) i.p. for 7 consecutive days (No. 2021050811). Then, mice of the sham group were fed with normal diet and then injected with normal saline (i.p.) for 7 consecutive days. The number of every group was six. The diagnostic efficacy of clinical indicators of FBXO45 was analyzed using the receiver operating characteristic (ROC) curve and the area under the curve.

Blood glucose levels of mice were measured using 16.7 mmol/l of blood glucose after 1 week as successful induction of diabetes. The mice were euthanized under anesthesia using 50 mg/kg pentobarbital sodium and sacrificed using strangulation. Their kidneys were collected for analysis after the induction of STZ at 4 months.

The lentiviral vectors carrying FBXO45 plasmid were designed and chemically synthesized by Hanyin Biotechnology Limited Company (Shanghai, China). FBXO45 plasmid (1 × 10^8^ TU) and administered into the mice through tail vein injection. Animals were approved by the Animal Care and Use Committee of our hospital.

### 2.3 Histological examination

Urine albumin and creatinine were measured by ELISA kit (Nanjing Jiancheng Bioengineering Research Institute, Nanjing, China). Kidney function (restricted kinematic [RKA]/remaining kidney volume indexed [RKVd]/Raman kidney volume [RKVm]/Raman kidney volumes [RKVs]) was measured using a Vevo 770 high-resolution imaging system (Visual Sonics, Canada) equipped with a high-frequency ultrasound probe (RMV-707B).

Kidney slices were dehydrated and paraffin embedded and Standard procedures were performed as literature [22]. Kidney tissue samples were fixed in 4% paraformaldehyde, paraffn-embedded, and then sectioned into 5 μM slices for PAS staining. Kidney tissue samples were observed using a fluorescence microscope (Zeiss Axio Observer A1, Germany). Kidney slices incubated with primary antibodies against collagen IV (1:200, CST, USA) overnight at 4°C with the appropriate secondary antibodies (1:200, Santa Cruz, CA, USA). Kidney tissue samples were observed using a fluorescence microscope (Zeiss Axio Observer A1, Germany).

### 2.4 Kidney function

Kidney function was measured using a Vevo 770 high-resolution imaging system (Visual Sonics, Canada) equipped with a high-frequency ultrasound probe (RMV-707B). Urine albumin and creatinine were measured on a spot urine sample with an ELISA kit (Nanjing Jiancheng Bioengineering Research Institute).

### 2.5 Lentivirus injection

The lentiviral vectors carrying FBXO45 plasmid were designed and chemically synthesized by Hanyin Biotechnology Limited Company (Shanghai, China). The constructs were diluted to a total volume of 200 μL (1 × 10^8^ TU of FBXO45 plasmid) and administered into the mice through tail vein injection.

### 2.6 Cell culture and treatment

Human renal tubular epithelial cells HK-2 cells were seeded in a culture dish with RPMI 1640 (Gibco) supplemented with 10% fetal bovine serum (Gibco) under a humidified 5% (v/v) CO_2_ atmosphere at 37°C. HK-2 cells were stimulated with 20 mmol/L d-glucose for the DN model for 24–72 h [23,24]. The transfections (FBXO45 plasmid, sc-417503, Santa Cruz Biotechnology, Inc.; si-FBXO45 plasmid, sc-78222, Santa Cruz Biotechnology, Inc.) were performed using Lipofectamine 2000 (Thermo Fisher Scientific, Shanghai, China). After 48 h of transfection, HK-2 cells were stimulated with 20 mmol/L d-glucose for 24–72 h.

### 2.7 ELISA assay, cell counting kit‑8 (CCK-8) assay, lactate dehydrogenase (LDH) activity, propidium iodide (PI) staining, and calcein/PI staining

Blood, tissue, or cell samples were collected and used to measure inflammation and oxidative stress levels using ROS production (S0033S), CAT (S0051), SOD (S0101S), MDA (S0131S), INF-γ (PI521), TNF-α (C1058S), IL-6 (PI330), and IL-1β (PI305) ELISA kits (Nanjing Jiancheng Biological Engineering Institute, Nanjing, China) following the manufacturer’s instructions. Cells were stained with DCFH-DA and were observed using a fluorescence microscope (Zeiss Axio Observer A1, Germany).

Calcein AM/CoCl2 assay and JC-1 disaggregation were evaluated by AM/CoCl2 assay Kit (C2009S, Beyotime Biotechnology, Nanjing, China) and JC-1 Kit (C2003S, Beyotime Biotechnology, Nanjing, China). For (CCK-8, C0037), 2 × 10^3^ cells/well were seeded in a 96-well plate.

LDH activity levels were assessed via the absorbance using a microplate reader (Thermo Fisher Scientific) at 450 nm. Cells were incubated with PI staining (ST512, Beyotime Biotechnology, Nanjing, China) or Calcein/PI staining (C2015S, Beyotime Biotechnology, Nanjing, China).

### 2.8 Quantitative polymerase chain reaction (qPCR)

Total RNAs were isolated with RNA isolator total RNA extraction reagent (Takara) and cDNA was synthesized using PrimeScipt RT Master Mix (Takara). qPCR was performed with the ABI Prism 7500 sequence detection system according to the Prime-Script™ RT detection kit. Relative levels of the sample mRNA expression were calculated and expressed as 2^−DDCt^.

### 2.9 Western blotting analysis

Membranes were incubated with SOX2 (ab92494, 1:2,000, Abcam), GPX4 (ab41787, 1:2,000, Abcam), FBXO45 (ab190688, 1:2,000, Abcam), PLK1 (ab189139, 1:2,000, Abcam), and β-Actin (BS6007MH, 1:5,000, Bioworld Technology, Inc.) at 4°C overnight. The membranes were incubated with horseradish peroxidase-conjugated secondary antibodies (sc-2004 or sc-2005, 1:5,000, Santa Cruz, USA) for 1 h at 37°C after washing with TBST for 15 min. Protein was measured using an enhanced chemiluminescence system (ECL, Beyotime) and analyzed using an Image Lab 3.0 (Bio-Rad Laboratories, Inc.).

### 2.10 Electron microscope and bioluminescence imaging

Tissues were fixed in 0.2 M phosphate buffer (KH_2_PO_4_/Na_2_HPO_4_, pH 7.5) supplemented with 2.5% glutaraldehyde (G5882, Sigma-Aldrich) for Electron microscope as literature. Ultra-thin sections were observed using a Hitachi H7650 transmission electron microscope (Tokyo, Japan).

HK-2-hPLK1-Luc was structured according to the previously described [25]. Bioluminescent imaging was performed using an IVIS imaging system (Bio-Real, QuickView3000, Austria).

### 2.11 Molecular docking

This study downloaded PLK1 (PDB ID: 4X9R) from the PBD database and downloaded the protein structure of FBXO45 from the Uniprot database. The protein structure was imported into Pymol 2.3.0 to remove crystal water and small molecules and then docked FBXO45 and PLK1 using Hdock. The docking results showed that the binding energy between FBXO45 and PLK1 was −246.99 kcal/mol. The residues around the protein–protein interaction interface can form hydrogen bonds. These non-covalent bonds can help stabilize protein–protein complexes. Pymol 2.3.0 is used to analyze the interaction mode of the docking results.

### 2.12 Statistical analysis

Data were expressed as mean ± standard error of the mean. Multiple comparisons were used GraphPad Prism 8 to perform by Student’s *t*-test or one-way analysis of variance followed by Tukey’s post-test. *P* values <0.05 were considered statistically significant.


**Ethical approval:** The current study was approved by the Ethics Committee of The Affiliated Shunde Hospital Of Jinan University. All procedures were performed in accordance with the Guidance Suggestions for the Care and Use of Laboratory experiments, formulated by the Ministry of Science and Technology of China.

## 3 Results

### 3.1 FBXO45 levels in model of DN

**Figure 1:**
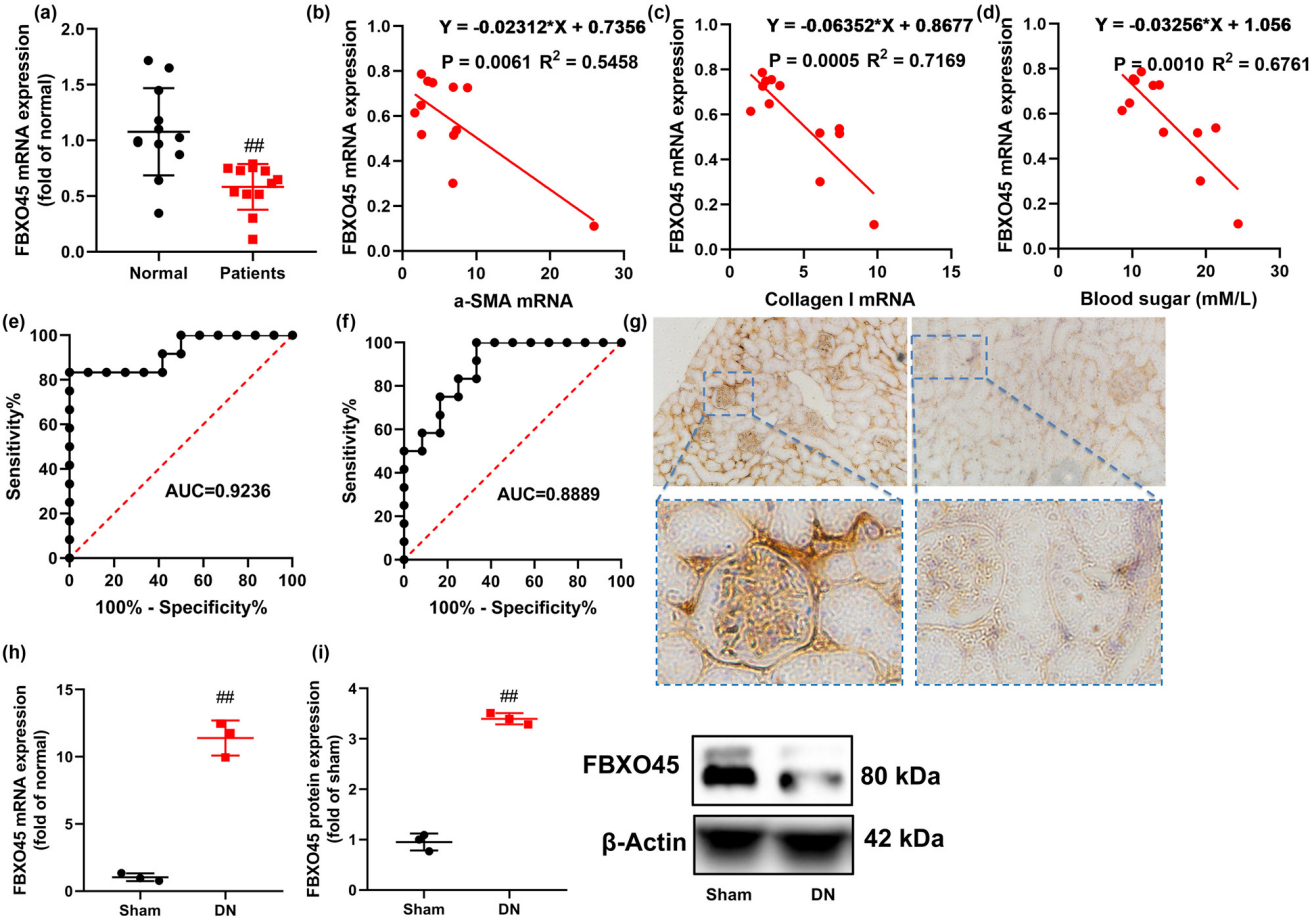
FBXO45 levels in model of DN. FBXO45 mRNA expression (a); FBXO45 mRNA expression was negative correlation with serum α-SMA (b), Collagen I (c) mRNA expression, blood sugar (d); ROC FBXO45 mRNA/proteinuria, (e and f) in patients with DN; FBXO45 expression (immunohistochemical, (g) bars = 100 μm), mRNA expression (h), and protein expression (i) in kidney tissue of mice model of DN. ^##^ *p* < 0.01 compared with the normal group or Sham group. The number of patients = 12, and the number of mice model = 6.

In this study, our primary objective was to investigate the levels of FBXO45 in a DN model. We observed that serum FBXO45 mRNA expression was significantly down-regulated in patients with DN (Figure 1a). Furthermore, we found a negative correlation between serum FBXO45 mRNA expression and the mRNA expression levels of α-SMA, Collagen I, and E-cadherin in patients with DN (Figure 1b–d). Moreover, we observed a negative correlation between serum FBXO45 mRNA expression and blood sugar levels, as indicated by the ROC value of 0.9236 (Figure 1e and f). To further validate these findings, we examined FBXO45 mRNA and protein expression in kidney tissue from a mice model of DN. Consistently, both FBXO45 mRNA and protein expression were reduced in the kidney tissue of DN mice (Figure 1g–i).

### 3.2 FBXO45 up-regulation reduced DN in mice model

**Figure 2:**
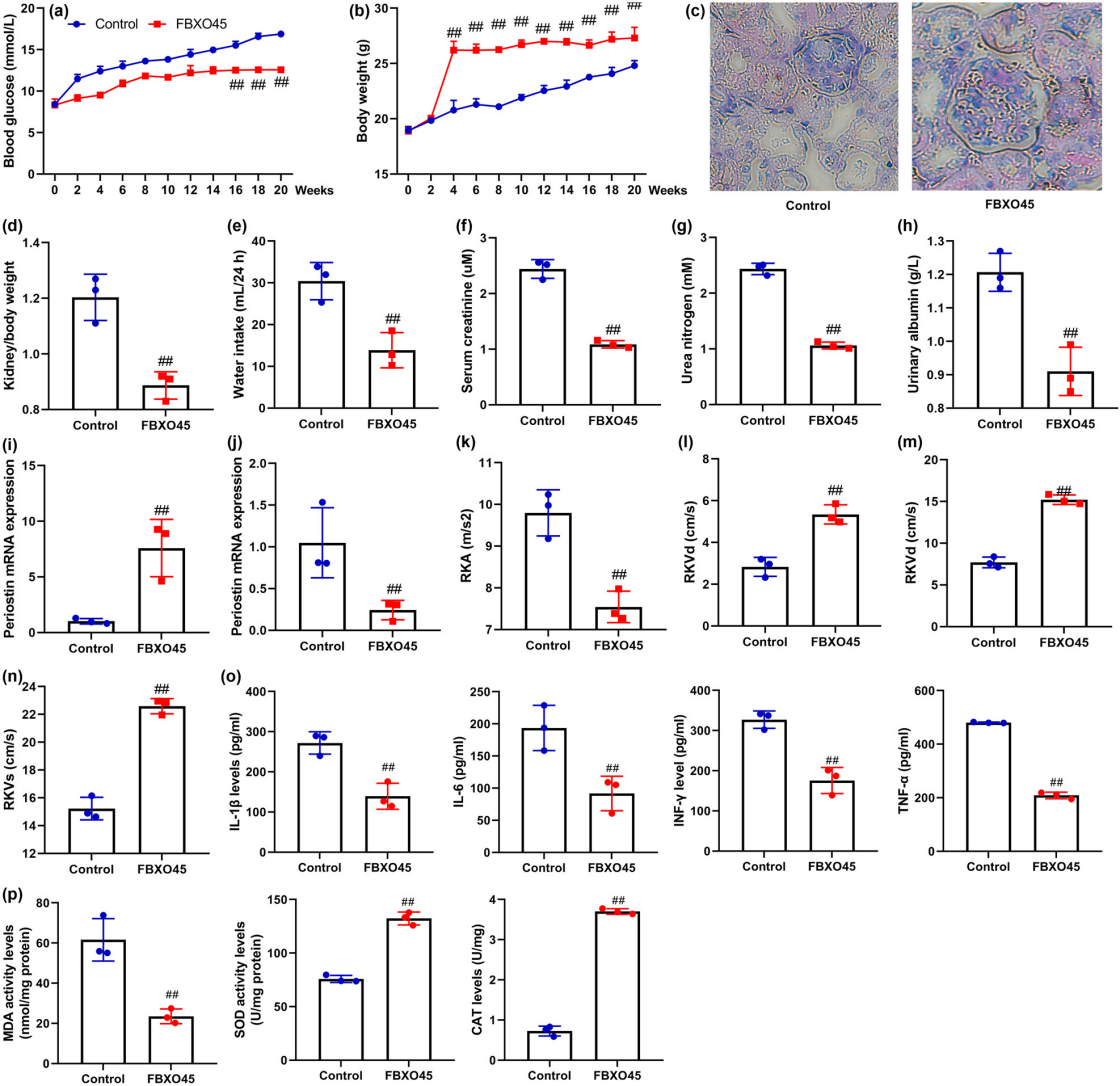
FBXO45 up-regulation reduced DN in mice model. FBXO45 mRNA expression (a), blood glucose (b), body weight (c), kidney/body weight (d), water intake 24 h (e), serum creatinine (f), urea nitrogen (g), Masson staining (h), urinary albumin levels (i), periostin mRNA expression (j), RKA/RKVd/RKVm/RKVs levels (k–n), and IL-1β/IL-6/INF-γ/TNF-α (o) and MDA/SOD/CAT (p) in mice model. The number of mice model = 6. ^##^ *p* < 0.01 compared with the control group. The number of vitro model = 3.

We conducted experiments using a mice model to examine the impact of FBXO45 on DN. FBXO45 lentiviral vectors increased the expression of FBXO45 mRNA in kidney tissue of DN mice (Figure 2a). Through the use of FBXO45 lentiviral vectors, we observed several beneficial effects. Specifically, the lentiviral vectors led to a reduction in blood glucose levels and kidney/body weight, while promoting an increase in body weight. Additionally, they contributed to improvements in glomerular structure, as evidenced by inhibited water intake and decreased serum creatinine levels. Moreover, we noted a decrease in urea nitrogen and urea nitrogen levels in the DN mice model (Figure 2b–h). Furthermore, the administration of FBXO45 lentiviral vectors resulted in increased periostin mRNA expression and RKA levels, along with a reduction in E-cadherin mRNA expression level, RKVd, RKVm, and RKVs levels in the mice model of DN (Figure 2i–n). Finally, the introduction of FBXO45 lentiviral vectors also yielded a reduction in inflammation and oxidative stress in the mice model of DN (Figure 2o–p). These findings suggest that FBXO45 has potential therapeutic benefits for alleviating the symptoms of DN.

### 3.3 FBXO45 up-regulation reduced inflammation and oxidative stress of Renal fibrocytes

**Figure 3:**
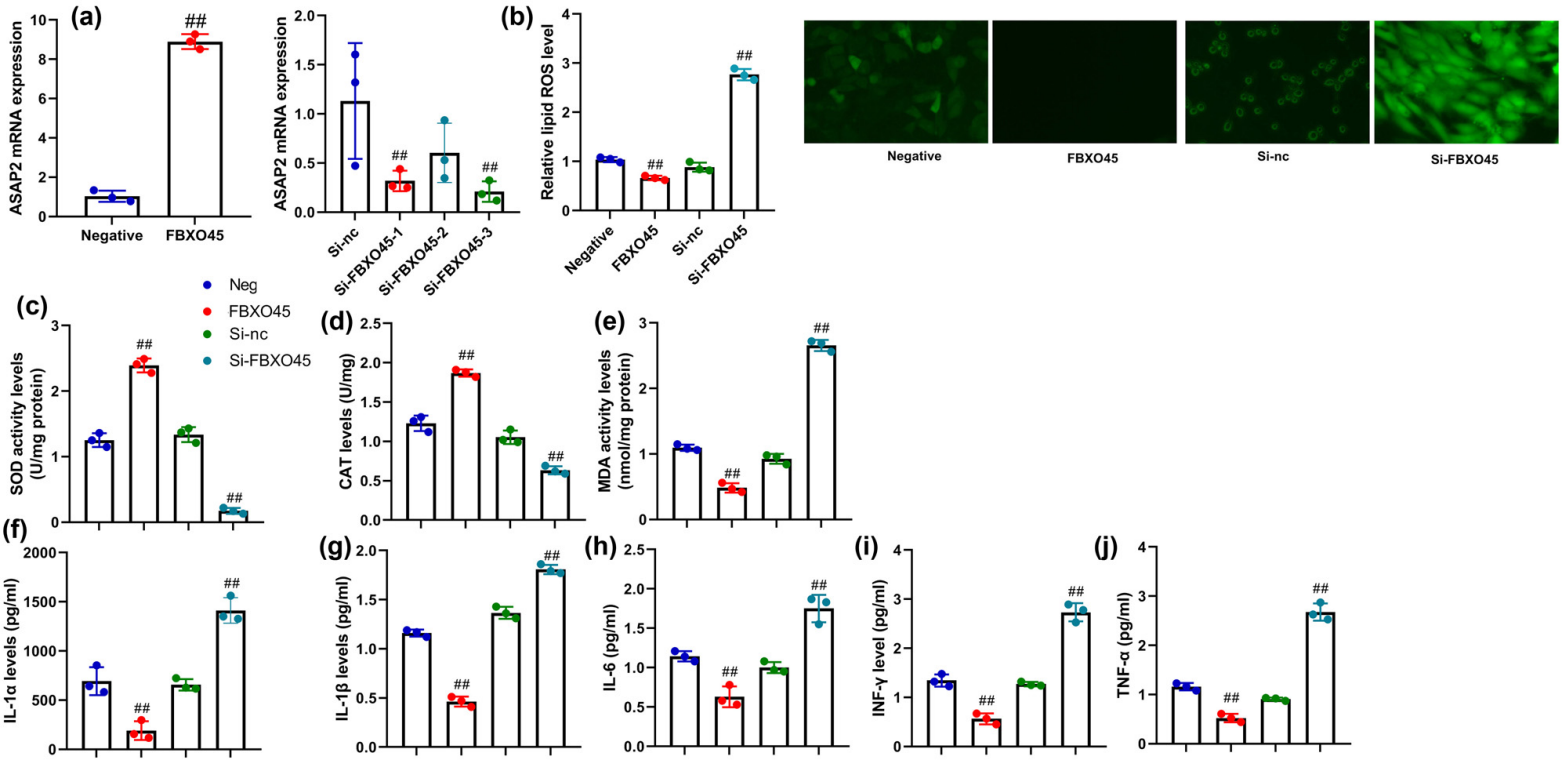
FBXO45 up-regulation reduced inflammation and oxidative stress of Renal fibrocytes. FBXO45 mRNA expression (a), lipid ROS level (b), SOD/CAT/MDA (c–e), and IL-1α/IL-1β/IL-6/INF-γ/TNF-α (f–j). Renal fibrocytes *in vitro* model. ^##^ *p* < 0.01 compared with negative or si-nc group. The number of vitro model = 3, and the number of mice model = 6.

This study aimed to investigate the impact of FBXO45 on inflammation and oxidative stress in renal fibrocytes. In an *in vitro* model of DN, we assessed the effects of FBXO45 plasmid and si-FBXO45 plasmid on FBXO45 mRNA expression in renal fibrocytes (Figure 3a). The results revealed that the introduction of FBXO45 plasmid led to an increase in FBXO45 mRNA expression, while the administration of si-FBXO45 plasmid resulted in a reduction of FBXO45 mRNA expression in renal fibrocytes. Furthermore, we examined the influence of FBXO45 up-regulation and down-regulation on ROS-induced oxidative stress and inflammation levels in the renal fibrocytes of the *in vitro* DN model (Figure 3b–j). Our findings showed that the up-regulation of FBXO45 contributed to a reduction in ROS-induced oxidative stress and inflammation levels in renal fibrocytes. Conversely, the down-regulation of FBXO45 led to an increase in ROS-induced oxidative stress and inflammation levels in these cells. These results suggest that FBXO45 plays a role in mitigating oxidative stress and inflammation in renal fibrocytes, highlighting its potential as a therapeutic target for managing complications associated with DN.

### 3.4 FBXO45 up-regulation reduced ferroptosis of renal fibrocytes

**Figure 4:**
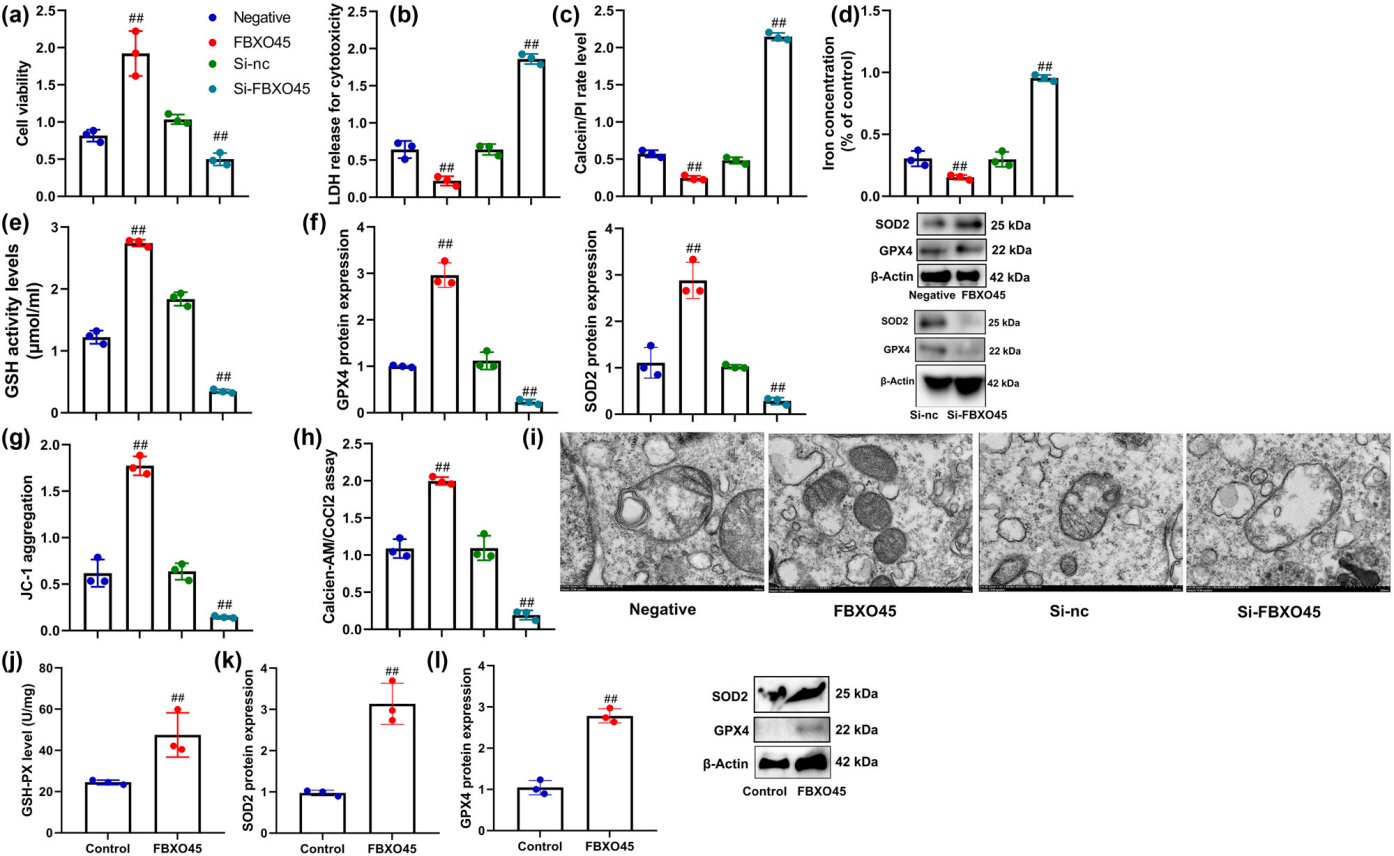
FBXO45 up-regulation reduced ferroptosis of renal fibrocytes. Cell viability (a), LDH activity level (b), proportions of PI-positive cells (c), iron concentration (d), GSH levels (e), GPX4 and SOD protein expression (f), JC-1 levels (g), MPT (h), mitochondrial damage (electron microscope) (i) *in vitro* model, GSH levels (j), and GPX4 and SOD protein expression (k and l). ^##^ *p* < 0.01 compared with negative or si-nc or control group.

In this study, we aimed to investigate the effects of FBXO45 on ferroptosis in renal fibrocytes within a model of DN. Through the over-expression of FBXO45, we observed increased cell growth and reductions in LDH levels, iron content, and PI-positive cells in renal fibrocytes subjected to the DN model (Figure 4a–d). Conversely, down-regulation of FBXO45 resulted in decreased cell growth, elevated LDH levels, iron content, and an increase in PI-positive cells in these cells (Figure 4a–d). Moreover, FBXO45 over-expression promoted GSH activity levels and induced the expression of GPX4 and SOD2 proteins in renal fibrocytes within the DN model (Figure 4e and f). Conversely, FBXO45 down-regulation led to the suppression of GPX4 and SOD2 protein expression and a reduction in GSH activity levels in renal fibrocytes subjected to the DN model (Figure 4e and f). Additionally, through FBXO45 over-expression, we observed increased JC-1 levels, reduction in mitochondrial permeability transition (MPT), and mitigation of mitochondrial damage in renal fibrocytes within the DN model (Figure 4g–i). Conversely, FBXO45 down-regulation resulted in reduced JC-1 levels, increased MPT, and promoted mitochondrial damage in these cells (Figure 4g–i). Furthermore, in the mice model, the administration of FBXO45 lentiviral vectors increased GSH activity levels and induced GPX4 and SOD2 protein expression within kidney tissue (Figure 4j–l). These findings suggest that FBXO45 plays a significant role in regulating ferroptosis in renal fibrocytes within the context of DN. Additionally, FBXO45 may contribute to the protection of kidney tissue through the modulation of GSH activity, GPX4, and SOD2 protein expression, as well as the preservation of mitochondrial integrity.

### 3.5 FBXO45 up-regulation induced PLK1 expression

**Figure 5:**
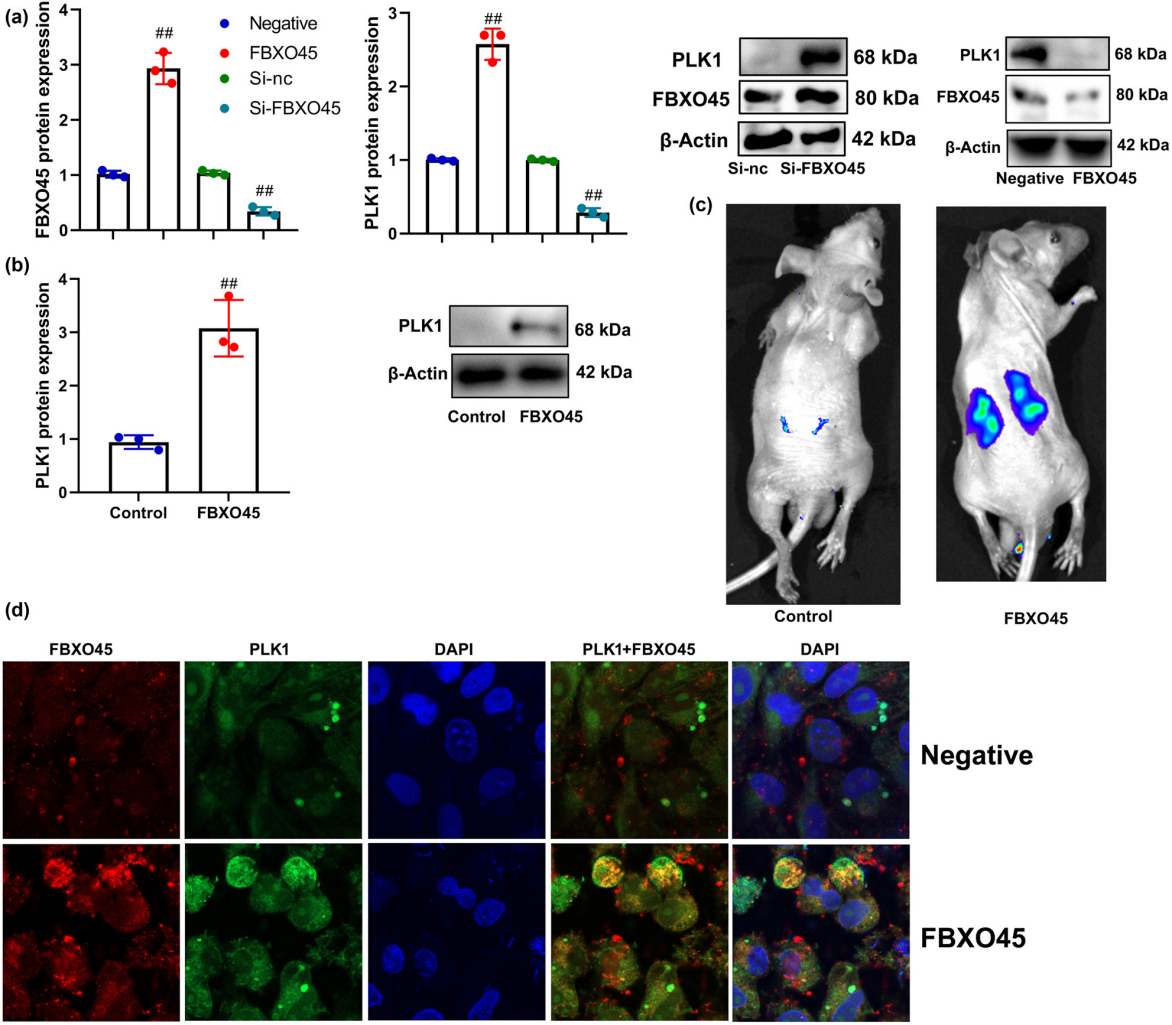
FBXO45 up-regulation induced PLK1 expression. FBXO45 and PLK1 protein expression *in vitro* model (a), PLK1 protein expression in mice model (b), bioluminescence imaging for PLK1 levels (c), and FBXO45 and PLK1 expression *in vitro* model immunofluorescence (d). ^##^ *p* < 0.01 compared with negative or si-nc or control group. The number of vitro model = 3, and the number of mice model = 6.

This study aimed to elucidate the underlying mechanism of FBXO45 in ferroptosis within a model of DN. Through the over-expression of FBXO45, we observed an induction of FBXO45 and PLK1 protein expression in renal fibrocytes subjected to the *in vitro* DN model (Figure 5a). Conversely, down-regulation of FBXO45 resulted in a reduction in FBXO45 and PLK1 protein expression in these renal fibrocytes (Figure 5a). Furthermore, in the mice model, the administration of FBXO45 lentiviral vectors also induced PLK1 protein expression in kidney tissue (Figure 5b and c). To further support these findings, immunofluorescence analysis demonstrated that FBXO45 over-expression increased the expression of FBXO45 and PLK1 in gastric cancer cells (Figure 5d). These results indicate that FBXO45 plays a crucial role in regulating the expression of FBXO45 itself and PLK1 in both renal fibrocytes and gastric cancer cells. These findings provide insights into the molecular mechanisms underlying the involvement of FBXO45 in the pathophysiology of DN and potentially other related diseases.

### 3.6 PLK1 inhibitor reduced the effects of FBXO45 on DN

**Figure 6:**
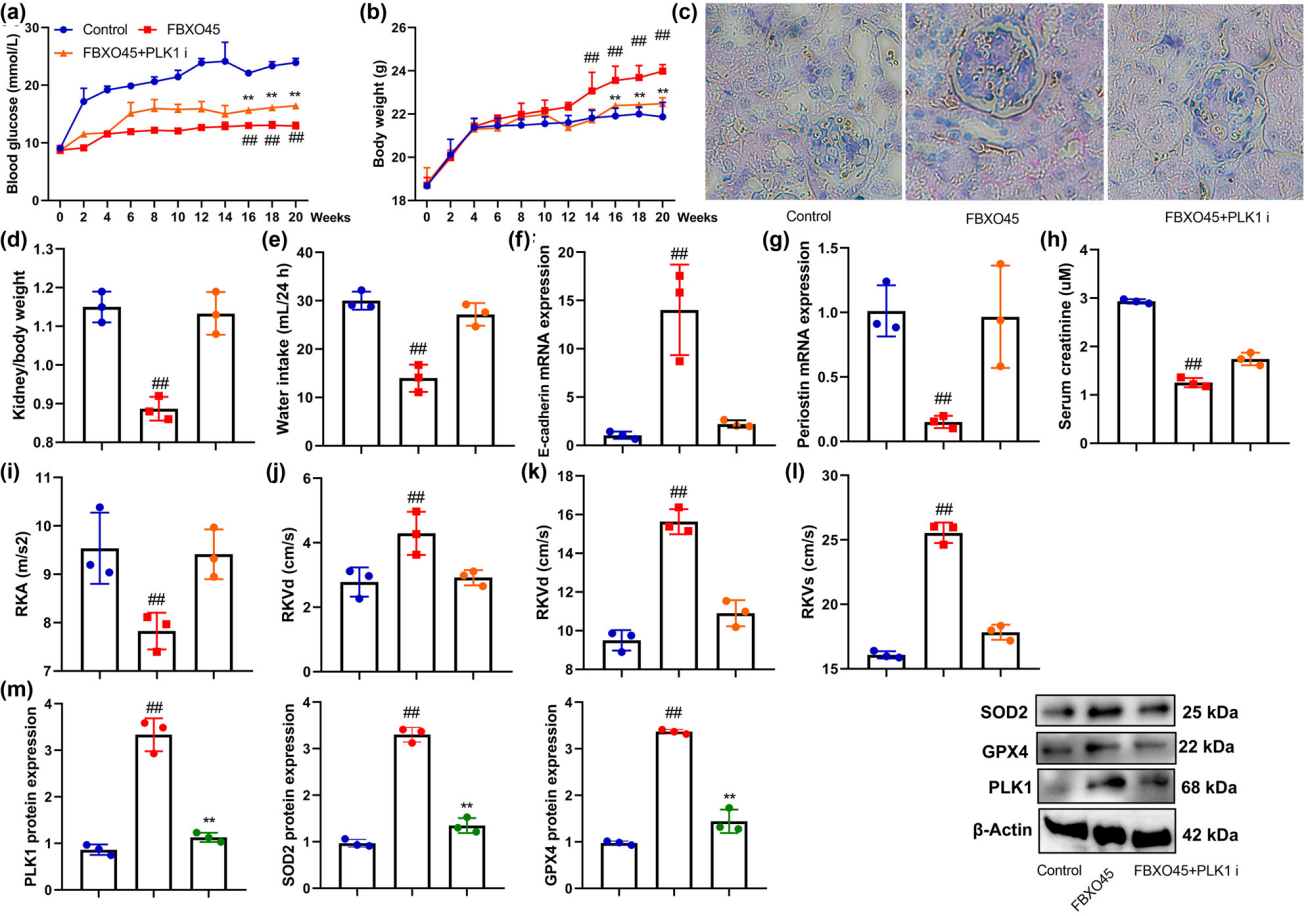
PLK1 inhibitor reduced the effects of FBXO45 on DN in the mice model. Blood glucose (a), body weight (b), PAS staining (c), kidney/body weight (d), water intake 24 h (e), E-cadherin mRNA expression (f), periostin mRNA expression (g), serum creatinine (h), RKA/RKVd/RKVm/RKVs levels (i–l), and PLK1/GPX4/SOD2 protein expression (m) *in vitro* model, ^##^ *p* < 0.01 compared with the control group; **p < 0.01 compared with FBXO45 group. The number of mice model = 6.

Moreover, we conducted further investigations to verify the involvement of PLK1 in the mechanism of FBXO45 within the DN model. To inhibit PLK1 activity, we utilized a PLK1 inhibitor called Onvansertib at a concentration of 10 μM. The results demonstrated that the administration of Onvansertib effectively mitigated the effects of FBXO45 on the expression of PLK1, GPX4, and SOD2, as well as on the development of DN in the mice model (Figure 6).

**Figure 7:**
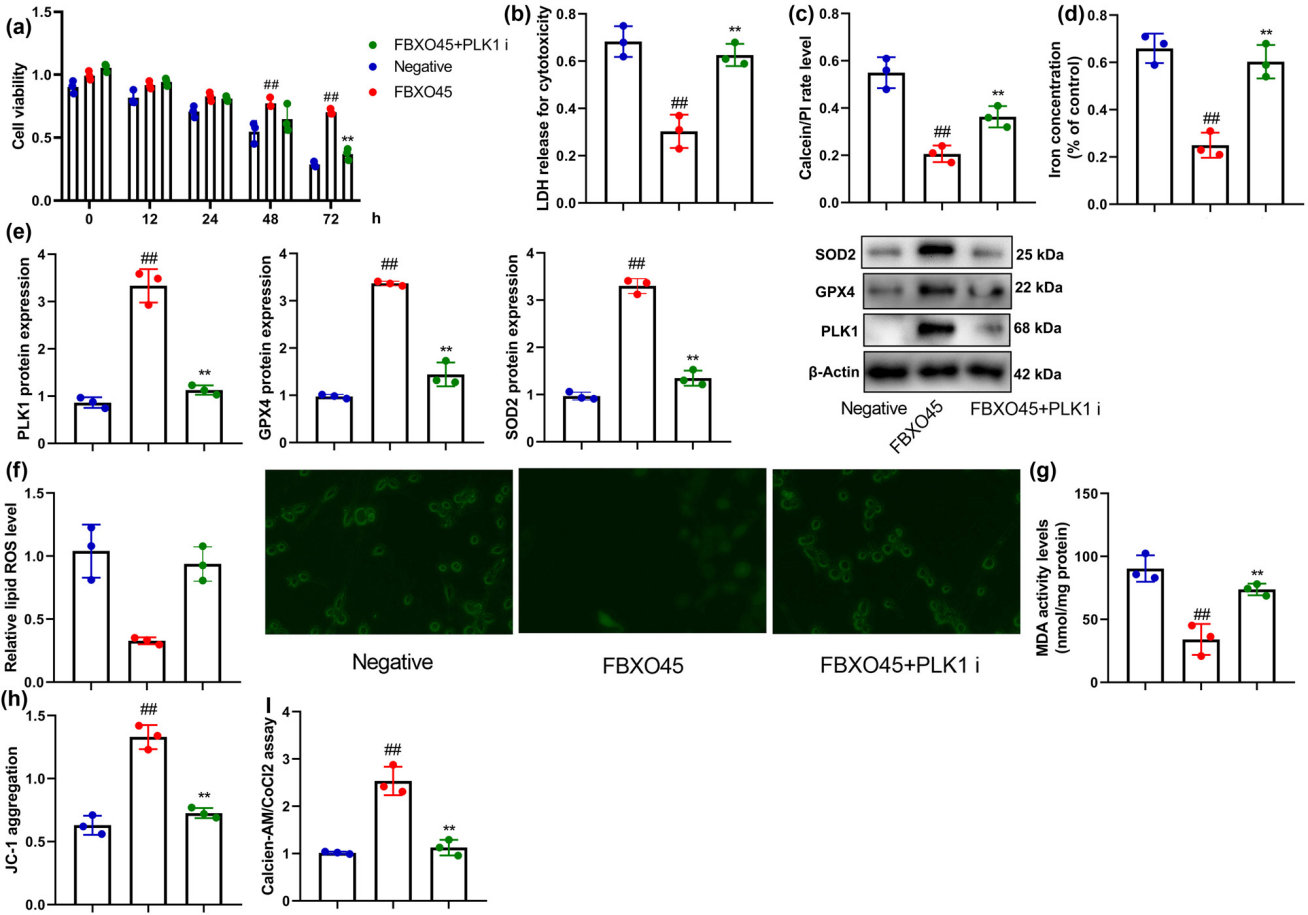
PLK1 inhibitor reduced the effects of FBXO45 on DN *in vitro* model. Cell viability (a), LDH activity level (b), proportions of PI-positive cells (c), lron concentration (d), PLK1/GPX4/SOD2 protein expression (e), lipid ROS level (f), MDA (g), JC-1 levels (h), and MPT (i). ^##^ *p* < 0.01 compared with the control group; **p < 0.01 compared with FBXO45 group. The number of vitro model = 3.

Furthermore, in an *in vitro* model of DN using renal fibrocytes, treatment with the PLK1 inhibitor also counteracted the effects of FBXO45 on PLK1, GPX4, SOD2 expression, and ferroptosis (Figure 7). These findings suggest that the mechanism of FBXO45 in the context of DN is mediated, at least partly, through its interaction with PLK1. The use of a PLK1 inhibitor effectively attenuated the impact of FBXO45 on the expression of PLK1, GPX4, SOD2, and the progression of ferroptosis both *in vivo* and *in vitro*. These results highlight the significance of the FBXO45-PLK1 axis in the pathogenesis of DN and offer potential therapeutic implications for targeting this pathway.

### 3.7 FBXO45 protein interlinked with PLK1 protein

**Figure 8:**
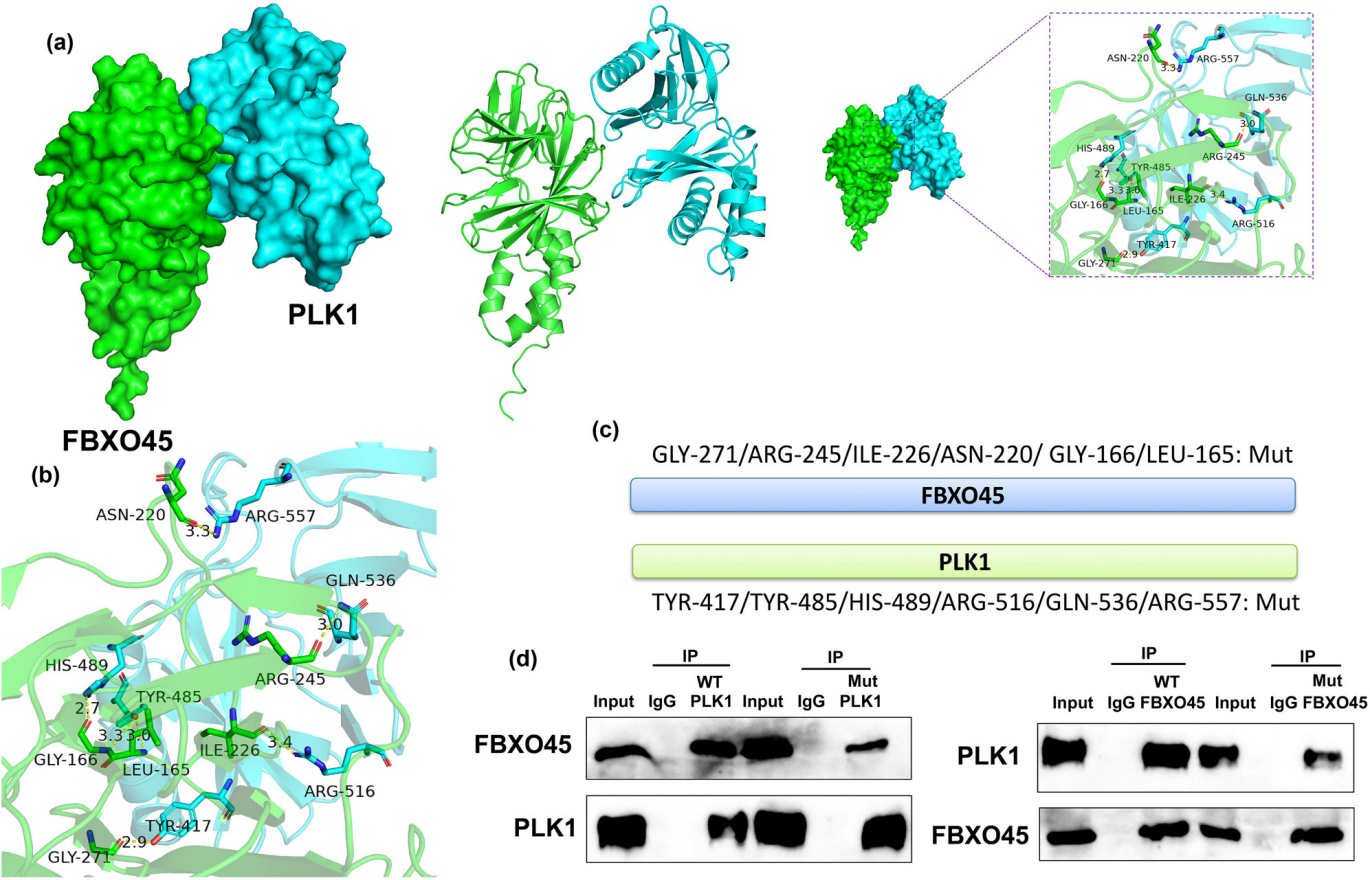
FBXO45 protein interlinked with PLK1 protein. 3D structure for FBXO45 protein interlinking with PLK1 protein (a), the binding site of TRIM47 protein interlinking with FOXO1 protein (b), schematic diagram of mutation sites (c), and IP assay for FBXO45 protein interlinking with PLK1 protein (d). The number of vitro model = 3.

In the subsequent phase of our study, we delved deeper into understanding the mechanism by which FBXO45 influences ferroptosis in renal fibrocytes. Using a 3D model prediction approach, we identified potential interaction sites between FBXO45 and PLK1. Specifically, the analysis revealed that FBXO45 residues GLY-271, ILE-226, GLY-166, LEU-165, ARG-245, and ASN-220 may interact with PLK1 residues TYR-417, ARG-516, HIS-489, TYR-485, GLN-536, and ARG-557 (Figure 8a and b). These predicted interactions provide valuable insights into the potential molecular interplay between FBXO45 and PLK1. To further validate this interaction, we constructed a schematic diagram highlighting the mutation sites and performed an immunoprecipitation (IP) assay. The results demonstrated the physical association between FBXO45 and PLK1 proteins, supporting the notion of direct interlinking between these two proteins (Figure 8c and d). These findings shed light on the molecular mechanisms underlying the influence of FBXO45 on ferroptosis in renal fibrocytes.

## 4 Discussion

Kidney disease is a prevalent clinical condition that has seen an increasing incidence of kidney injury attributed to diabetes, owing to the rapid socioeconomic development, improved living standards, and changing lifestyles in China [26]. Diabetes is a group of chronic endocrine and metabolic disorders characterized by persistently elevated blood glucose levels. When the disease progresses, it can lead to kidney damage [27], eventually resulting in DN, which is a major microvascular complication of diabetes and the primary cause of end-stage renal disease [28]. In our study, we observed a down-regulation of serum FBXO45 mRNA expression in patients with DN as well as in a mouse model of DN. FBXO45 prevents renal fibrocytes in the model of DN through the inhibition of ferroptosis by PLK1 activity. These findings align with the work by Chuang et al., who reported decreased Fbxo45 expression in neuronal processes [29]. Collectively, these data provide evidence suggesting the regulatory role of Fbxo45 in DN-induced renal fibrocytes. The identification of FBXO45 as a potential player in the pathogenesis of DN underscores the importance of elucidating the molecular mechanisms underlying this disease. Further investigations into the precise role of FBXO45 in mediating renal fibrocyte function may offer valuable insights for the development of targeted therapeutic strategies aimed at ameliorating the progression of DN. α-SMA, COL I, and E-cad, these indicators are correlated with FBXO45. Of course, these indicators are not typical secret proteins, which is also a limitation of this study. We will continue to investigate the correlation between FBXO45 and DN in the next experiment.

Diabetes is a prevalent and increasingly common disease [30]. As people’s living standards improve and lifestyles change, the number of individuals diagnosed with diabetes continues to rise, along with the associated kidney damage. In China, diabetes nephropathy has surpassed glomerulonephritis as the leading cause of chronic kidney disease among hospitalized patients [31]. The etiology and pathogenesis of diabetes nephropathy are multifaceted, involving various factors such as genetic predisposition, renal hemodynamic effects, inflammatory responses, metabolic disruptions due to hyperglycemia, hypertension, abnormal metabolism of vasoactive substances, oxidative stress, and more. It is worth noting that the early stages of diabetes nephropathy often feature subtle signs, such as negative urinary albumin excretion, which can easily go unnoticed [32]. Our study provides data demonstrating the up-regulation of FBXO45 in a mouse model, which resulted in reduced DN. Interestingly, Lin et al. have previously suggested that FBXO45 could be targeted by miR-485-3p to modulate neuroinflammation in Parkinson’s disease [33]. These findings collectively suggest that FBXO45 may hold potential for the treatment of renal fibrocytes in the context of DN. By shedding light on the role of FBXO45 in mitigating the effects of DN, our research contributes to the understanding of this complex disease and highlights FBXO45 as a potential therapeutic target. Further investigations into the underlying molecular mechanisms and pathways involved may lead to the development of novel interventions for the treatment of DN.

The progression of interstitial fibrosis, a challenging condition to treat, is a prominent characteristic of progressive renal disease (PRD) and a major contributor to poor prognosis and limited treatment options for PRD patients in clinical settings [34]. Although many kidney diseases originate from the glomerulus, the extent of involvement of the renal tubulointerstitium serves as the most reliable prognostic indicator. PRD can have various causes, including obesity, hypertension, diabetes, or rare gene mutations. While its precise mechanism remains unclear, the ultimate outcome typically involves aggravated RF and eventual destruction of the blood-filtering cells [35]. In our study, we observed that up-regulation of FBXO45 led to a reduction in inflammation and oxidative stress in renal fibrocytes. This finding aligns with the work by Hsieh et al., who demonstrated that TNF-α suppresses Fbxo45 expression, contributing to the development of neuropathic allodynia in rats [36]. These data suggest that FBXO45 may have the potential to alleviate inflammation and oxidative stress in models of DN. Our findings provide insights into the role of FBXO45 in mitigating inflammation and oxidative stress in renal fibrocytes. Understanding the molecular mechanisms underlying these effects could pave the way for novel therapeutic strategies targeting RF in PRDs. Further investigations are warranted to elucidate the downstream pathways and molecular interactions involved in FBXO45-mediated modulation of inflammation and oxidative stress in the context of DN.

Ferroptosis is a distinct form of cell death that differs from apoptosis, necrosis, autophagy, and other known types of cell death [37]. The term “ferroptosis” was first proposed in 2012 [38]. It is an iron-dependent regulated cell death process driven by oxidative damage to polyunsaturated fatty acid-containing phospholipids in the cellular membrane. This process involves three key steps: iron accumulation, iron-dependent ROS production, and lipid peroxidation [39]. In our study, we observed that up-regulation of FBXO45 led to a reduction in ferroptosis in renal fibrocytes in both a mouse model of DN and an *in vitro* DN model. These findings are consistent with the work by Wang et al., who demonstrated that Fbxo45 inhibited apoptosis in Kyse-150, Kyse30, and ECA-109 cells [40]. Collectively, these data suggest that FBXO45 plays a role in reducing ferroptosis in renal fibrocytes within the context of DN. The identification of FBXO45 as a potential modulator of ferroptosis in renal fibrocytes provides new insights into the pathogenesis of DN. Further investigation into the underlying molecular mechanisms and signaling pathways involved may uncover novel therapeutic targets for preventing or treating ferroptosis-associated RF in the context of DN.

PLK1 is a member of the Polo-like kinase family, which comprises serine/threonine kinases found widely in eukaryotic cells [41]. Through its kinase activity, PLK1 can modulate various substrates, thereby regulating processes such as cell mitosis, cytokinesis, DNA damage response, and development [42]. High expression levels of PLK1 have been observed in several tumor tissues, including breast cancer, prostate cancer, ovarian cancer, and neuroblastoma [43]. In our study, we demonstrate that up-regulation of FBXO45 induces the expression of PLK1, and FBXO45 protein interacts with PLK1 protein in a model of DN. These findings align with the work by Lin et al., who reported the involvement of FBXO45 in liver tumorigenesis through upregulation of PLK1 [14]. Collectively, these data suggest that FBXO45 interacts with PLK1 protein to enhance the activity level of PLK1 in the context of DN. The identification of the interaction between FBXO45 and PLK1 sheds light on their potential collaborative role in the pathogenesis of DN. Further investigations into the underlying mechanisms governing this interaction may provide valuable insights into the development of targeted therapeutic approaches aimed at modulating PLK1 activity in the treatment of DN.

## 5 Conclusion

In conclusion, our study demonstrates that FBXO45 plays a crucial role in preventing renal fibrocytes in a model of DN through its regulation of PLK1 activity. These findings uncover a novel tissue-protective function of FBXO45 and highlight its potential therapeutic implications for targeting ferroptosis-associated renal fibrocytes in the context of DN. The identification of FBXO45 as a modulator of PLK1 activity provides new insights into the underlying molecular mechanisms involved in the pathogenesis of DN. Further exploration of the therapeutic potential of targeting FBXO45 in the context of ferroptosis-related RF may pave the way for the development of innovative treatments for DN. Overall, our study expands our understanding of the complex regulatory network involved in DN and suggests FBXO45 as a promising candidate for future therapeutic interventions aimed at mitigating the progression of this debilitating disease.
